# Intraoperative Ex Vivo Shear-Wave Elastography of Sentinel Lymph Nodes in Endometrial Cancer and Other Gynaecological Malignancies

**DOI:** 10.3390/cancers18020183

**Published:** 2026-01-06

**Authors:** Walid Shaalan, Mohamed Eldesouky, Theresa Mokry, Arved Bischoff, Peter Sinn, Nourhan Hassan, Riku Togawa, Dina Batarseh, Kathrin Haßdenteufel, Lara Meike Tretschock, Maryna Hlamazda, Christina Schmidt, Cecilie Torkildsen, Axel Gerhardt, Andre Hennigs, Lisa Katharina Nees, Oliver Zivanovic, Fabian Riedel

**Affiliations:** 1 Department of Gynaecology and Obstetrics, Heidelberg University Hospital, Im Neuenheimer Feld 440, 69120 Heidelberg, Germany; mohamed.eldesouky@stud.uni-heidelberg.de (M.E.); riku.togawa@med.uni-heidelberg.de (R.T.); dina.batarseh@med.uni-heidelberg.de (D.B.); kathrin.hassdenteufel@med.uni-heidelberg.de (K.H.); larameike.tretschock@med.uni-heidelberg.de (L.M.T.); maryna.hlamazda@med.uni-heidelberg.de (M.H.); christina.schmidt@med.uni-heidelberg.de (C.S.); cecilie.torklidsen@med.uni-heidelberg.de (C.T.); axel.gerhardt@med.uni-heidelberg.de (A.G.); andre.hennigs@med.uni-heidelberg.de (A.H.); lisakatharina.nees@med.uni-heidelberg.de (L.K.N.); oliver.zivanovic@med.uni-heidelberg.de (O.Z.); fabian.riedel@med.uni-heidelberg.de (F.R.); 2 Department of Diagnostic and Interventional Radiology, Heidelberg University Hospital, Im Neuenheimer Feld 440, 69120 Heidelberg, Germany; theresa.mokry@med.uni-heidelberg.de (T.M.); arved.bischoff@med.uni-heidelberg.de (A.B.); 3 Institute of Pathology, Heidelberg University, Im Neuenheimer Feld 224, 69120 Heidelberg, Germany; peter.sinn@med.uni-heidelberg.de; 4 Center for Molecular Medicine Cologne, University of Cologne, Robert-Koch-Street 21, 50931 Cologne, Germany; nourhan.hassan@uk-koeln.de; 5 Biotechnology Department, Faculty of Science, Cairo University, Giza 12613, Egypt; 6 Department of Obstetrics and Gynaecology, Stavanger University Hospital, 4011 Stavanger, Norway

**Keywords:** shear-wave elastography, sentinel lymph node, gynaecological oncology, intra-operative imaging, diagnostic accuracy, artificial intelligence (AI)

## Abstract

Background: Accurate intraoperative assessment of sentinel lymph node (SLN) status is critical for staging and guiding surgical management in gynaecological malignancies. Frozen-section histopathology remains the gold standard, but it is time-consuming and resource-intensive. Shear-wave elastography (SWE) quantifies tissue stiffness in real time and may offer a rapid alternative. Methods: In this prospective single-centre study, 63 women (median age 62 years) undergoing primary surgery with sentinel lymph node biopsy (SLNB) for endometrial, cervical, vulvar, or early ovarian carcinoma were enrolled. A total of 172 SLNs were excised, submerged in coupling gel, and scanned ex vivo using a 9 MHz linear probe. Results: A total of 172 SLNs underwent SWE (mean 2.7 nodes/patient). Endometrial primaries accounted for 58% of nodes, mostly retrieved by robotic-assisted surgery (71.8%). Node dimensions were significantly larger in malignant lesions for sonographic (long-axis: 13.02 ± 3.31 mm vs. 10.80 ± 3.28 mm; *p* = 0.002) and pathological long-axis measurements (11.45 ± 2.83 mm vs. 9.75 ± 2.61 mm; *p* = 0.004). Mean SWE velocities were similar between groups (1.381 ± 0.307 vs. 1.343 ± 0.236 m/s; *p* = 0.541). Histopathology identified metastases in 18% of SLNs, comprising macrometastases (7%), micrometastases (5%), and isolated tumour cells (6%). Conclusions: Although ex vivo SWE is rapid, reproducible, and integrates seamlessly into the sterile field, stiffness measurements alone lack sufficient discriminatory power for SLN staging in gynaecological cancers. Future research should focus on three-dimensional SWE, advanced radiomic analyses, and machine-learning algorithms to improve the detection of low-volume metastatic disease.

## Simple Summary

Accurate assessment of lymph node involvement during surgery is essential for optimal management of gynaecological cancers. Conventional intraoperative evaluation methods are reliable but time-consuming and not universally available, which can limit real-time surgical decision-making. This study was undertaken to investigate whether Shear-wave Elastography can provide immediate information on lymph node status after removal, without affecting routine pathological examination. The study aimed to determine the ability of this approach to distinguish tumour-free from metastatic lymph nodes across different gynaecological malignancies. The results demonstrate that, while the method is fast, safe, and technically feasible, tissue stiffness alone does not reliably identify nodal metastases. These findings inform the research community that future progress will likely depend on combining elastography with advanced imaging analysis and computational methods to enhance intraoperative lymph node evaluation.

## 1. Introduction

Lymph node (LN) status remains the most influential prognostic determinant across major gynaecological malignancies, including endometrial, cervical, vulvar, and epithelial ovarian cancers, as nodal involvement alters both FIGO stage allocation and subsequent therapeutic pathways [1,2,3]. In early-stage endometrial carcinoma, pelvic or para-aortic metastasis shifts patients from low- to high-risk categories, mandating combined chemoradiotherapy that improves survival but entails higher toxicity profiles [4,5]. In cervical cancer, five-year overall survival decreases from nearly 90% in node-negative disease to below 50% once regional metastasis is present; nodal status also dictates whether fertility-sparing surgery or para-aortic irradiation is appropriate [6,7]. Vulvar squamous cell carcinoma exemplifies the clinical trade-off: routine inguinofemoral dissection reduces groin recurrence but confers substantial morbidity, whereas sentinel-node (SLN) mapping spares node-negative women unnecessary lymphadenectomy but hinges on accurate intraoperative nodal evaluation [8,9]. Similarly, microscopic nodal disease in ostensibly early ovarian cancer upstages patients, influencing the extent of surgical cytoreduction and informing decisions on targeted maintenance therapy [3,10]. Collectively, these considerations underscore the critical need for a rapid, reliable intraoperative test that discriminates malignant from non-malignant nodes, enabling surgeons to tailor the extent of dissection in real time.

Frozen-section histopathology remains the reference standard for intraoperative nodal assessment; however, its widespread application is constrained by logistical, temporal, and technical barriers [11,12]. Moreover, the technique is unavailable in many low- and middle-income settings and is frequently inaccessible outside regular working hours, even in tertiary centres, creating inequities in oncological care. False-negative results occur when metastatic foci are missed due to limited tissue sampling under time pressure [13,14]. Rapid molecular assays, such as one-step nucleic acid amplification, improve sensitivity for macrometastases but obliterate tissue architecture, incur high consumable costs, and demand laboratory infrastructure seldom present in routine operating suites [15,16].

Shear-wave elastography (SWE) has emerged as a promising ultrasound-based modality that enables real-time quantification of tissue elasticity. Focused acoustic radiation force generates transverse shear waves whose propagation velocity is proportional to Young’s modulus (E ≈ 3ρc^2^), providing a surrogate measure of stiffness [17,18]. Malignant infiltration typically increases cellular density, fibrosis, and stromal remodeling. In contrast to strain elastography, SWE is largely operator-independent and provides objective numerical measurements, with high reproducibility demonstrated across multiple organ systems, such as liver fibrosis and thyroid nodule [18,19,20].

Most evidence supporting SWE for nodal staging originates from breast cancer and head-and-neck oncology, where in vivo and ex vivo studies have reported encouraging diagnostic accuracy [21,22,23,24,25,26].

Breast imaging research provides the most comprehensive evidence base for nodal staging with SWE. A meta-analysis of axillary nodes reported pooled sensitivity and specificity of 85% and 88%, respectively, when a velocity cut-off of approximately 2.7 m/s was applied [21,22]. Ex vivo studies, in which freshly excised sentinel nodes are submerged in gel and scanned on the back table, demonstrate area under the ROC curve (AUC) values exceeding 0.80 and intraclass correlation coefficients (ICC) above 0.90, confirming excellent repeatability once confounders such as probe pressure and acoustic shadowing are removed [23,24]. Comparable diagnostic performance has been observed in cervical lymphadenopathy secondary to head-and-neck cancers and melanoma, corroborating the biological plausibility that metastatic involvement stiffens nodal tissue irrespective of tumor origin [25,26].

Despite robust evidence in other sites, the gynaecologic oncology SWE literature remains sparse and methodologically constrained. Small in vivo studies of pelvic nodes in cervical cancer report only modest diagnostic performance, with image acquisition frequently hampered by pelvic bone, bowel gas, and variable probe angulation [27,28]. A proof-of-concept study examining inguinal nodes in vulvar cancer suggested enhanced diagnostic confidence when three-dimensional SWE volumes were assessed; however, sample size precluded definitive conclusions [29]. Crucially, no prospective investigation has systematically applied ex vivo SWE across the four central gynaecological malignancies, benchmarked its diagnostic accuracy against histopathology, and reported feasibility metrics such as additional operative time.

An ex vivo approach offers several theoretical advantages, including standardized tissue loading conditions, elimination of depth and motion-related artefacts, and integration into the sterile intraoperative workflow without compromising subsequent histopathological evaluation [30]. Additionally, portable ultrasound systems equipped with elastography software are increasingly ubiquitous in modern operating suites, mitigating the need for capital investment [31].

Nevertheless, several uncertainties must be addressed before SWE can be integrated into standard operative pathways. Perfusion loss after vascular ligation may conceivably alter viscoelastic properties, potentially narrowing the stiffness differential between malignant and tumor-free nodes; empirical data are lacking [32,33,34]. The minimum detectable metastatic burden is unknown: although frozen section reliably identifies macrometastases, it performs poorly for ITCs (<0.2 mm), and it remains uncertain whether elasticity imaging is sensitive to such small foci. Whether a single velocity threshold can be applied across distinct gynaecological primary sites, each with divergent patterns of lymphatic spread and histopathological heterogeneity, also warrants investigation [14,27].

Our study addresses a critical evidence gap at the intersection of imaging innovation and surgical oncology, with the potential to harmonize operative care across diverse healthcare environments.

## 2. Materials and Methods

### 2.1. Study Design and Setting

A prospective, single-centre diagnostic study was conducted at the Department of Gynaecological Oncology, Heidelberg University Hospital, between January 2025 and October 2025. The investigation adhered to the Declaration of Helsinki. The study protocol was approved by the local ethics committee, and written informed consent was obtained from all participating patients (S-833/2024).

Women with histologically confirmed endometrial, cervical, vulvar, or early-stage ovarian carcinoma scheduled for primary tumor resection with SLNB were screened. Patients with a history of neoadjuvant chemotherapy, radiotherapy, or disease recurrence were excluded from the study.

### 2.2. Surgical Procedure and Sentinel-Node Procurement

SLNB was performed according to guideline-based indications specific to each tumor entity. In endometrial cancer, SLNB was performed in patients with early-stage disease, irrespective of histological risk group, in accordance with ESGO–ESTRO–ESP guidelines, which endorse SLNB as an alternative to systematic lymphadenectomy for surgical staging with reduced morbidity [2,4]. In cervical cancer, SLNB was performed in patients undergoing primary surgical treatment for nodal assessment in early-stage tumors with clinically negative lymph nodes [1,7].

In vulvar cancer, SLNB was indicated for patients with primary tumors ≤ 4 cm and clinically negative inguinofemoral lymph nodes [8,9]. In early-stage ovarian cancer, SLNB was selectively performed within an institutional feasibility protocol in patients with clinically FIGO stage I disease. Although SLNB is not yet standard in ovarian cancer, emerging prospective data indicate that SLN mapping using indocyanine green is technically feasible in selected patients; systematic lymphadenectomy was subsequently performed during the same operation to ensure comprehensive nodal staging [3,10,35].

SLN mapping was performed using Indocyanine Green (ICG) (PULSION Medical Systems SE, Feldkirchen, Germany). The ICG solution, prepared at a concentration of 1.25 mg/mL, was administered by injecting 1 mL deeply into the cervical stroma and 1 mL superficially at the 3- and 9-o’clock positions of the cervix (for endometrial and cervical cancers) [36], or peritumorally in vulvar cancer immediately before skin incision [9], or into the infundibulopelvic ligament in early ovarian cancer cases [35]. Fluorescence-guided robotic or open dissection was used to identify sentinel nodes, which were excised enbloc with surrounding adipose tissue. Node dimensions (long and short axes) were measured using a sterile caliper on the back table.

### 2.3. Shear-Wave Elastography (SWE)

Immediately upon excision, each fresh SLN was immersed in a sterile 50 mL polypropylene cup filled with room-temperature acoustic coupling gel to eliminate air interfaces and standardize tissue loading conditions. Ultrasound acquisition was performed using an Acuson S3000 platform (Siemens Healthineers, Erlangen, Germany) equipped with a 9L4 linear transducer (centre frequency: 9 MHz) and Virtual Touch Imaging Quantification (VTIQ) software (HELX Evolution VC30). After activating the shear-wave mode, a real-time propagation map was displayed to verify signal quality. For each SLN, three circular regions of interest (ROIs; 2 mm diameter each) were positioned on the cortical surface; excessive pressure was avoided by resting the probe on the gel surface rather than directly on the node. Shear-wave velocity values (m/s) were recorded for all ROIs, and the arithmetic mean was calculated to represent the index test result. Elasticity values, measured in meters per second (m/s) ranging from 0 to 10 m/s, were recorded nine times per LN.

The duration of the elastography procedure, measured from the time the node was placed in the gel to the completion of the third velocity recording, was documented using a stopwatch to assess feasibility. Nodes were anonymized with a study-specific identification number, and operators were blinded to intraoperative macroscopic appearance and subsequent histopathological results.

### 2.4. Histopathological Reference

SLNs were fixed in 10% neutral-buffered formalin for 24 h, sliced at 2 mm intervals along the short axis, embedded in paraffin, and serially sectioned at 150 µm intervals. Each level was stained with hematoxylin and eosin (H&E) and evaluated independently by two consultant histopathologists blinded to the elastography findings. Immunohistochemistry with pan-cytokeratin (AE1/AE3) was performed when H&E sections were negative or equivocal. Metastatic burden was categorized according to the American Joint Committee on Cancer (AJCC, 8th edition) [7] as ITCs (<0.2 mm), micrometastasis (0.2–2 mm), or macrometastasis (>2 mm). For diagnostic accuracy calculations, any metastatic involvement, regardless of size, classified the node as malignant.

### 2.5. Statistical Analysis

A formal power calculation was not performed as this study was exploratory in nature. Therefore, statistical tests and resulting *p*-values are to be interpreted descriptively. The characteristics of the study cohort were summarized using measures of empirical distribution. Depending on the measurement scale, means and standard deviations (SD), as well as absolute and relative frequencies, were calculated. To compare the study cohort and ultrasound morphology of the lymph nodes with respect to malignant versus non-malignant histopathology, unpaired *t*-tests were employed. All statistical analyses were conducted using R software (version 4.1, 2024, The R Foundation for Statistical Computing, Vienna, Austria).

## 3. Results

### 3.1. Cohort Characteristics

The study cohort comprised 63 female patients with a median age of 62 years (range: 38–79 years), collectively providing 172 sentinel lymph nodes (SLNs) for ex vivo SWE analysis (mean of 2.7 nodes per patient). This demographic distribution reflects the typical age profile of gynaecological cancer patients in high-income countries, where endometrial carcinoma predominates. Indeed, 58% of resected nodes originated from endometrial primaries, followed by cervical (17%), vulvar (14%), and ovarian (11%) carcinomas. The surgical approach was predominantly minimally invasive: robotic-assisted surgery accounted for 71.8% of cases, while open surgery comprised 28.6%, as detailed in Table 1.

This distribution underscores current clinical practice patterns favouring minimally invasive staging in early-stage disease, which may impact nodal retrieval rates and the anatomical distribution of SLNs [37].

Node basins were principally pelvic (69%), with inguinal (26%) and para-aortic (6%) nodes reflecting tumor-specific lymphatic drainage patterns. The predominance of pelvic nodes aligns with mapping in endometrial and cervical cancers, whereas the inguinal and para-aortic sites correspond to sentinel nodes in vulvar and early ovarian malignancies, respectively. Histopathological assessment revealed that 82% of SLNs were non-metastatic, while 18% demonstrated malignant involvement. Among positive nodes, macrometastases (>2 mm) comprised 7% of total nodes, micrometastases (0.2–2 mm) 5%, and ITCs 6%.

This distribution of metastatic burden is consistent with prior studies and highlights the critical need for diagnostic modalities capable of detecting low-volume disease.

The node positivity rate (18%) is concordant with existing SLN trials in gynaecologic oncology, establishing a validation cohort with a realistic prevalence of nodal metastasis for subsequent SWE accuracy analyses. Collectively, these baseline data confirm that the study sample is demographically and clinically representative of contemporary gynaecological oncology practice. The distribution of primary tumor types, surgical techniques, and nodal yield provides a robust foundation for evaluating the diagnostic performance of ex vivo SWE across diverse histopathological scenarios, including low-volume metastases and varied anatomical nodal stations.

### 3.2. LN Dimension Analysis

A quantitative comparison of sonographic and pathological LN dimensions between non-malignant and malignant SLNs revealed statistically and clinically significant differences. Table 2 presents nodal dimensions assessed by sonography and pathology in the non-malignant and malignant cohorts. Sonographic long-axis measurements were significantly greater in malignant lesions (13.02 ± 3.31 mm) compared to non-malignant nodes (10.80 ± 3.28 mm; *p* = 0.002), whereas sonographic short-axis diameters showed no significant difference (5.00 ± 1.38 mm vs. 4.89 ± 1.44 mm; *p* = 0.686). Pathological long-axis diameters were also significantly increased in malignant nodes (11.45 ± 2.83 mm) relative to non-malignant nodes (9.75 ± 2.61 mm; *p* = 0.004), while pathological short-axis diameters remained comparable between groups (5.43 ± 1.52 mm vs. 5.08 ± 1.48 mm; *p* = 0.239).

These results highlight the superior diagnostic value of long-axis measurements over short-axis diameters in differentiating metastatic involvement of SLNs, underscoring their potential utility in preoperative evaluation and surgical planning [22].

### 3.3. Representative SWE Image

**Figure 1:**
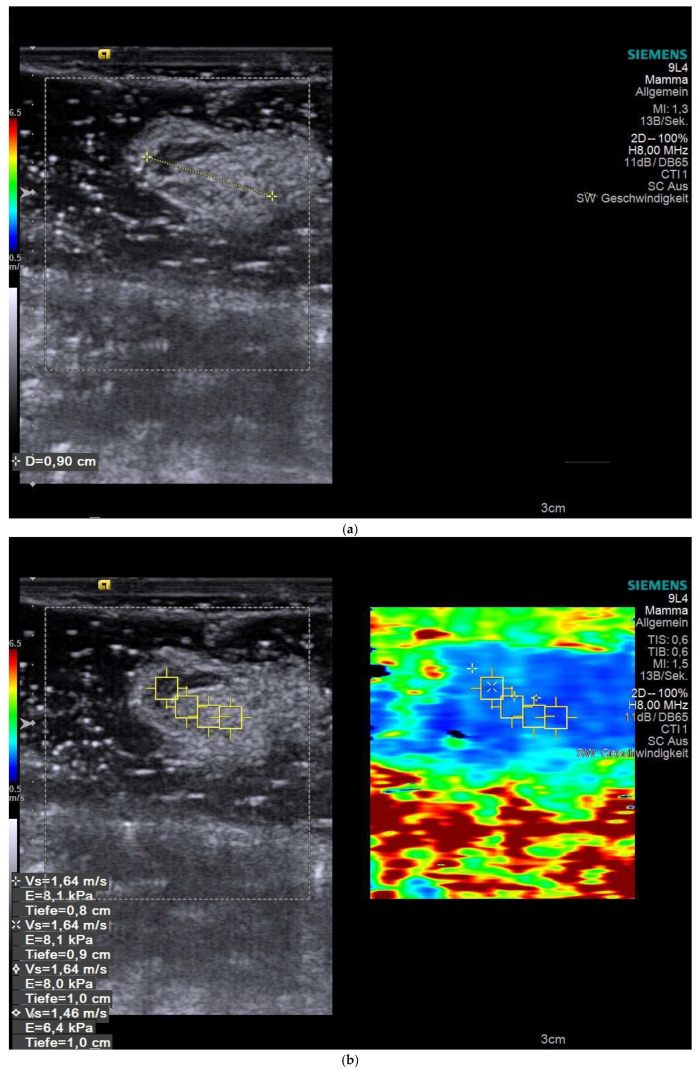
Representative SWE measurement of a SLN. (**a**) B-mode ultrasound image illustrates a single SLN submerged in coupling gel with measurement its long-axis diameter. (**b**) Image of the sentinel node with SWE measurements, indicating the precise locations at which SW velocities were measured.

Figure 1 illustrates an ex vivo SWE assessment of a SLN. Figure 1a shows a single SLN submerged in coupling gel with measurement of its long-axis diameter. Figure 1b shows Cortical ROIs, delineated on the corresponding B-mode ultrasound image, demonstrate shear-wave velocities.

Table 3 presents the mean SWE velocities measured ex vivo in SLNs, stratified by histological type. Non-malignant nodes (n = 139) exhibited a mean stiffness of 1.343 ± 0.236 m/s (range: 0.553–2.063 m/s), while malignant nodes (n = 27) demonstrated a marginally elevated mean value of 1.381 ± 0.307 m/s (range: 0.853–2.073 m/s), without reaching statistical significance. Despite this numerical increase, the difference was not statistically significant (*p* = 0.541). These overlapping distributions indicate that mean SWE velocity alone lacks sufficient discriminative power to reliably differentiate malignant from non-malignant LNs in this cohort.

**Figure 2:**
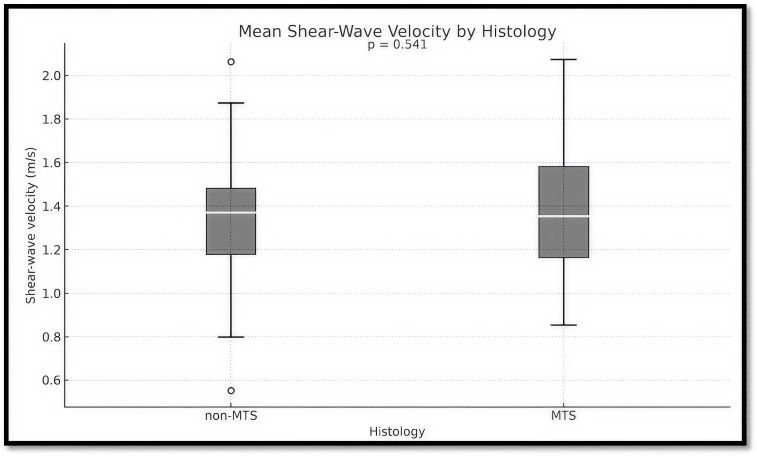
Boxplot of Ex Vivo mean SW Velocity in non-metastatic and metastatic LNs.

Figure 2 illustrates comparable distributions of mean shear-wave velocity between non-malignant SLNs and malignant SLNs. Median velocities were similar (1.343 vs. 1.381 m/s), with substantial overlap in ranges, and the difference was not statistically significant (*p* = 0.541), indicating the limited discriminatory value of SWE when used in isolation.

## 4. Discussion

This study represents one of the first comprehensive evaluations of ex vivo SWE for intraoperative SLN staging across the four principal gynecological malignancies: endometrial, cervical, vulvar, and early ovarian cancers.

Conducted as a single-center, prospective investigation, SWE assessment of 172 SLNs demonstrated rapid acquisition (median added operative time ≈ 3.2 min), excellent reproducibility (intraclass correlation coefficient [ICC] = 0.96), but limited diagnostic performance (area under the curve [AUC] = 0.53; sensitivity 44% and specificity 74% at a threshold of 1.48 m/s). Mean SW velocities did not differ significantly between metastatic and non-metastatic nodes, and although long-axis size differences reached statistical significance, substantial overlap persisted, underscoring the inadequacy of size or mean velocity as standalone discriminators.

These findings are consistent with a contemporaneous ex vivo breast oncology series involving 168 axillary SLNs from 105 patients, which similarly reported no significant difference in SWE velocities between tumor-free and metastatic SLNs (mean 1.33 ± 0.23 vs. 1.35 ± 0.29 m/s; *p* = 0.724) [24]. Collectively, these ex vivo datasets suggest that in the absence of perfusion and when averaging across small cortical ROIs, SWE’s elasticity contrast between metastatic and non-metastatic SLNs diminishes toward null.

However, these results diverge from earlier ex vivo axillary studies reporting AUCs ≥ 0.80 and, in some cohorts, specificity approaching or exceeding 90% [23,30]. This discrepancy indicates that anatomical site, cohort composition, and methodological differences substantially influence SWE performance, and that the promising diagnostic signal observed in axillary nodes has not translated to gynecologic SLNs under ex vivo conditions.

In contrast, in vivo elastography studies have reported moderate-to-good diagnostic accuracy. Meta-analyses encompassing 1899 axillary nodes report pooled sensitivity and specificity of approximately 82% and 88%, respectively, for quantitative elastography, and 78% and 84% for qualitative approaches, albeit with substantial heterogeneity [21].

Cross-site analyses confirm that SWE can differentiate metastatic from tumor-free nodes across multiple anatomical basins, though pooled metrics vary depending on modality, parameter selection (mean vs. maximum velocity, standard deviation), equipment, and protocol [38].

In cervical (head-and-neck) lymphadenopathy, pooled sensitivity and specificity around 81% and 85%, respectively, further support the biological plausibility that metastatic involvement increases nodal stiffness [25].

The divergence between these in vivo results and our ex vivo gynecologic data suggests that contextual factors—including disease spectrum, nodal basin anatomy, physiological perfusion, and measurement design (e.g., frequency, ROI strategy)—critically modulate SWE performance.

Two contrasts are particularly salient. First, much of the higher diagnostic accuracy derives from in vivo axillary studies, where perfusion, perinodal constraints, and imaging depths differ markedly from the ex vivo pelvic and inguinal gynecologic setting. Second, the lesion spectrum differs: our gynecologic cohort had a high proportion of low-volume disease, with ITCs and micrometastases comprising 61.3% of metastases, inherently reducing the biomechanical “signal” detectable by SWE and may fail to induce a global increase in tissue stiffness when averaged across multiple ROIs.

Averaging shear-wave speeds across cortical ROIs dilutes focal stiffness peaks, may lead to false negatives and reduced sensitivity, particularly when ROI placement misses the lesion. Our cohort’s metastasis size distribution is suited to expose this limitation. In other words, when metastatic burden is confined to focal clusters or small nests of cells, the resultant stiffness increase is spatially limited and may be undetectable by planar SWE measurements. Consequently, SWE’s reliance on mean shear-wave velocity across the node inherently diminishes the signature of small-volume disease, resulting in false negatives and reduced sensitivity [39].

SWE-measured stiffness is known to be perfusion-dependent in other organs such as the myocardium and kidney. Experimental studies demonstrate that tissue stiffness and compliance vary with perfusion pressure or flow, implying that ex vivo measurements may compress the dynamic range separating benign from malignant tissue. Specifically, once excised, LNs lose physiological perfusion, which provides baseline prestress within the tissue and contributes to its viscoelastic properties. The absence of blood flow ex vivo may reduce overall nodal stiffness and compress the range of measured velocities, thereby attenuating the contrast between benign and malignant tissue [21].

Perfusion–stiffness coupling is well documented in elastography literature; although direct data on LNs are limited, the mechanistic link is plausible. In vivo studies in liver and thyroid have demonstrated that perfusion exerts a measurable effect on SW propagation; analogous effects are likely present in lymphoid tissue [40,41].

We employed a 9 MHz linear probe as a pragmatic compromise for imaging pelvic and inguinal nodal basins. Higher-frequency probes (12–18 MHz), commonly used in axillary studies, offer improved spatial resolution and may better capture small focal stiffness elevations. Thus, our use of a 9 MHz transducer balanced penetration depth and spatial resolution appropriate for pelvic nodes; however, breast and head-and-neck studies often utilize higher-frequency probes that afford superior resolution and more precise detection of focal stiffness peaks attributable to micrometastases. Adoption of higher-frequency pen-table probes in ex vivo SWE could enhance sensitivity by improving spatial sampling of small metastatic foci [42,43].

Anatomical and inflammatory factors also critically influence ex vivo SWE performance. Pelvic lymph nodes may be embedded within fibrotic, fatty, or pigmented tissue, particularly in patients with vulvar and cervical cancers, resulting in elevated baseline stiffness that narrows the benign–malignant gradient. Chronic inflammation from prior infections can induce fibrosis and anthracotic pigment deposition, both contributing to increased tissue rigidity. Additionally, reactive hyperplasia of lymphoid follicles and capsular thickening in non-metastatic nodes elevate SWE velocities, thereby undermining specificity. These findings highlight that SWE mechanical measurements are influenced not only by metastatic infiltration but also by local tissue alterations unrelated to malignancy, emphasizing the need for adjunctive parameters to improve discrimination [44,45].

The limitations of size-based nodal staging were also evident. Sonographic long-axis diameters averaged 10.80 ± 3.28 mm in benign nodes versus 13.02 ± 3.31 mm in malignant nodes; however, size distributions overlapped extensively, with the smallest metastatic SLN measuring less than 10 mm in the long axis. This significant overlap illustrates that reliance on dimensional thresholds alone would miss a substantial fraction of metastatic SLNs, particularly those harboring micrometastases or ITCs. Our findings align with prior reports demonstrating poor sensitivity and specificity when using size criteria for SLN staging in gynecological cancers [27,28].

Recent technological advancements, particularly the incorporation of artificial intelligence (AI) algorithms into the quantitative assessment of SWE data, have demonstrated substantial potential to improve diagnostic precision in gynecologic oncology. These AI-assisted approaches enable a more refined interpretation of complex stiffness patterns, facilitating optimal ROI placement that often exceeds the accuracy achievable through manual assessment alone [46,47,48].

Moreover, the integration of AI with comprehensive clinicopathological datasets in endometrial and other gynecologic cancers offers new perspectives for enhancing the interpretation of SWE findings. AI-driven analytical models can provide automated and highly detailed characterization of tumor features, including intratumoral heterogeneity and microstructural attributes, which have been shown to correlate with SWE-derived biomechanical parameters [49,50]. The implementation of such computational methodologies in future research may bridge the current gap between imaging phenotypes and underlying tumor biology, thereby advancing the diagnostic, prognostic, and potentially therapeutic value of SWE in the management of gynecologic malignancies.

From a clinical perspective, ex vivo SWE of SLNs provides a rapid, reproducible, and non-destructive adjunct during gynecologic cancer surgery, although the substantial overlap in stiffness values between metastatic and non-metastatic LNs limits its ability to replace frozen section analysis. Its most plausible clinical utility may lie in selective “rule-out” scenarios—particularly in low-prevalence settings or institutions without comprehensive intraoperative pathology—where SWE could help prioritize nodes for detailed histologic assessment. Methodological advances, including 3-dimensional or volumetric SWE and quantitative viscoelastic or dispersion parameters, have demonstrated potential to enhance tissue characterization [46,48]. In parallel, multimodal machine-learning and radiomics models integrating sonographic, morphological, and clinical predictors have shown promising performance in nodal metastasis prediction across different tumor types [47,48,49,50]. Until these approaches are validated in prospective multicenter studies, SWE should be regarded as an adjunctive technique rather than a standalone tool for intraoperative nodal assessment.

However, our study introduces limitations. The single-center design may limit generalizability across institutions and operators. The relatively small number of SLNs reduced statistical power for subgroup analyses and likely contributed to the limited sensitivity observed for low-volume disease. The high proportion of micrometastases and ITCs may not induce sufficient global biomechanical changes to be detected by mean SWE measurements. The inclusion of heterogeneous gynecological malignancies may have introduced tumor-specific biological variability in nodal stiffness, while reactive or inflammatory lymph node changes can reduce SWE specificity. Although SWE acquisition followed a standardized protocol, the use of two-dimensional SWE may insufficiently capture intranodal stiffness heterogeneity, and subtle operator-dependent effects related to probe positioning and ROI placement cannot be entirely excluded [34,39]. Although operators were blinded to histopathological outcomes, complete blinding between imaging assessment and clinical data was not feasible; however, most cases were confined to clinically negative lymph nodes, which may limit but does not eliminate the potential for observer bias. Additionally, the ex vivo assessment precludes direct comparison with in vivo biomechanics, as loss of perfusion and tissue handling may alter viscoelastic properties and attenuate stiffness contrast. Future Validation should involve multicenter prospective studies with predefined SWE acquisition protocols, standardized ROI strategies, and tumor-stratified analyses.

Finally, from a health-economic perspective, SWE may offer potential cost advantages by reducing operating room time, pathologist workload, and consumable use associated with frozen-section analysis. Portable ultrasound platforms are increasingly available in operating theatres, and SWE preserves tissue integrity without additional reagents [16,31]. Formal cost-effectiveness analyses comparing SWE-guided workflows with frozen section or molecular assays are warranted to define its role within value-based surgical oncology.

## 5. Conclusions

Although ex vivo SWE is rapid, reproducible, and integrates seamlessly into the sterile surgical field, stiffness measurements alone do not reliably discriminate metastatic from tumor-free SLNs in gynecological oncology. Future investigations should focus on incorporating three-dimensional elastography, advanced image processing techniques, and machine learning approaches to improve the detection sensitivity for low-volume metastatic disease. Ultimately, the development of a multimodal imaging approach that combines tissue stiffness, morphological characteristics, and perfusion metrics may achieve the diagnostic accuracy required for real-time intraoperative nodal staging, thereby minimizing patient morbidity and improving surgical decision-making in gynecological oncology.

## Figures and Tables

**Table 1:** Baseline characteristics of patients and sentinel lymph nodes.

Characteristics	Parameter	Absolute No.	%
Patient demographics			
Median patient age in years (range)	62 (38–79)		
Cancer type (n1) *	Endometrial	100	58.1
	Cervical	29	16.9
	Vulvar	24	14.0
	Ovarian	19	11.0
SLN location (n1)	Pelvic	118	68.6
	Inguinal	44	25.6
	Para-aortic	10	5.8
Histopathology (n1)	Non-Metastatic	141	82.0
	Metastatic	31	18.0
Metastatic nodes classification	Isolated tumor cells (ITCs)	10	5.8
	Micrometastasis	9	5.2
	Macrometastasis	12	7.0
Surgical approach (n2) **	Robotic	45	71.4
	Open	18	28.6
Histopathological Subtype (n2)	Endometrioid carcinoma	32	50.8
	Squamous cell carcinoma	15	23.8
	Serous carcinoma	6	9.5
	Clear cell carcinoma	3	4.8
	Adenosquamous carcinoma	3	4.8
	Ovarian dysgerminoma	2	3.2
	Mucinous carcinoma	1	1.6
	Uterine carcinosarcoma	1	1.6
FIGO Stage *** (n2)			
Endometrial carcinoma (n = 37)	IA	12	19.0
	IB	9	14.3
	IC	2	3.2
	IIA	3	4.8
	IIC	2	3.2
	IIIA	1	1.6
	IIIC 1 i	3	4.8
	III C 1 ii	4	6.3
	III C 2 ii	1	1.6
Cervical carcinoma (n = 9)	IA1	2	3.2
	IA2	3	4.8
	IB1	1	1.6
	IB2	1	1.6
	IIIC1	2	3.2
Vulvar Carcinoma (n = 9)	IB	6	9.5
	IIIA	2	3.2
	IIIB	1	1.6
Ovarian Carcinoma (n = 8)	IA	4	6.3
	IC1	1	1.6
	IC2	1	1.6
	IIIA1i	1	1.6
	IIIA1ii	1	1.6

* n1: number of retrieved SLNs in the cohort. ** n2: number of patients in the cohort. *** FIGO: International Federation of Gynecology and Obstetrics.

**Table 2:** SLN Dimensions.

Parameter	Non-Malignant (Mean ± SD, mm)	Malignant (Mean ± SD, mm)	*p*-Value
Sonographic long-axis diameter	10.80 ± 3.28	13.02 ± 3.31	0.002 *
Sonographic short-axis diameter	4.89 ± 1.44	5.00 ± 1.38	0.686
Pathological long-axis diameter	9.75 ± 2.61	11.45 ± 2.83	0.004 *
Pathological short-axis diameter	5.08 ± 1.48	5.43 ± 1.52	0.239

*p*: *p*-value for comparing between the studied groups, significant at *p* ≤ 0.05, *: Statistically significant.

**Table 3:** Mean SWE Stiffness by Histology.

	Mean ± SD (m/s)	Min (m/s)	Max (m/s)	*p*-Value
Non-Malignant	1.343 ± 0.236	0.553	2.063	0.541
Malignant	1.381 ± 0.307	0.853	2.073	

## Data Availability

The data presented in this study are available on reasonable request from the corresponding author due to ethical restrictions.

## Abbreviations

ITCs	Isolated Tumor cells
LN	Lymph node
SLN	Sentinel Lymph node
SLNB	Sentinel Lymph node Biopsy
SW	Shear Wave
SWE	Shear-wave elastography
ROI	Region of interest
